# Real-world effectiveness of remdesivir in immunocompromised patients hospitalized due to SARS-CoV-2 Infection: Insights to inform pharmacy practice

**DOI:** 10.1093/ajhp/zxag036

**Published:** 2026-02-09

**Authors:** Andre C Kalil, Chidinma Chima-Melton, Emi Naslazi, Heribert Ramroth, Alpesh N Amin, Natasha N Pettit, Robert L Gottlieb

**Affiliations:** Division of Infectious Diseases, Department of Internal Medicine, University of Nebraska Medical Center, Omaha, NE, USA; Cardiopulmonary Division, Pipeline Health, Los Angeles, CA; Tele-ICU Inc., Los Angeles, CA, USA; Evidence and Access, Certara, The Netherlands; Medical Affairs, Gilead Sciences, Foster City, CA, USA; Department of Medicine, School of Medicine, University of California Irvine, Irvine, CA, USA; Department of Pharmacy, UChicago Medicine, Chicago, IL, USA; Department of Internal Medicine, Baylor University Medical Center, Baylor Scott & White Health, Dallas, TX; Burnett School of Medicine at TCU, Fort Worth, TX, USA

**Keywords:** antiviral therapy, COVID-19, early remdesivir, hospital pharmacy, immunocompromised, mortality

## Abstract

**Purpose:**

Immunocompromised adults hospitalized for coronavirus disease 2019 (COVID-19) remain at high risk for morbidity. Despite remdesivir’s approval for treatment of COVID-19, real-world evidence on its early use, particularly in patients infected with evolving Omicron-era subvariants, remains limited. Contemporary evidence is essential to support hospital pharmacy practice and antiviral stewardship.

**Methods:**

We conducted a retrospective cohort study using the Premier Healthcare Database. Adult immunocompromised patients hospitalized for COVID-19 from December 2021 to December 2024 were included. Patients with COVID-19 present-on-admission who received remdesivir within the first 2 hospital days were compared to untreated patients using 1:1 propensity score matching. The outcomes assessed were 14- and 28-day all-cause mortality. Subgroup analyses assessed outcomes stratified by oxygen requirements and specific immunocompromising conditions.

**Results:**

Among 22,808 matched patients, early remdesivir use was associated with significantly lower 14-day (hazard ratio [HR], 0.75; 95% CI, 0.69-0.82; *P* < 0.0001) and 28-day mortality (HR, 0.80; 95% CI, 0.74-0.86; *P* < 0.0001). Mortality reductions were observed across early and later Omicron periods and among patients with or without supplemental oxygen requirements. Subgroup analyses showed similar survival benefit in patients with cancer, including hematologic malignancies, as well as transplant recipients.

**Conclusions:**

Early remdesivir initiation was associated with clinically meaningful and significant survival benefits in immunocompromised patients hospitalized for COVID-19, with these associations persisting in the Omicron subvariant era. These findings highlight the potential benefit of early antiviral treatment in this vulnerable population while underscoring the need for further prospective studies, including randomized controlled trials, to confirm causal effects and refine treatment strategies. Clinical pharmacists have a pivotal role in ensuring institutional protocols remain aligned with current evidence so that eligible high-risk patients consistently receive appropriate therapy.

Key points

- Early inpatient remdesivir use is associated with significantly lower 14- and 28-day mortality among immunocompromised adults hospitalized for COVID-19.
- These findings offer timely real-world evidence to inform clinical pharmacy and antiviral stewardship in this high-risk population.

Immunocompromised individuals represent a growing and vulnerable group within the US population. According to the National Health Interview Survey, approximately 6.6% of US adults live with some form of immunosuppression.^1^ This group has experienced a disproportionate burden of coronavirus disease 2019 (COVID-19)–related adverse outcomes, and a relative excess burden endures. Data from COVID-NET (March 2020 to February 2022) show immunocompromised patients made up 12.2% of adult COVID-19 hospitalizations, far exceeding their population share.^2^ More recent surveillance data from COVID-NET show that approximately 15.6% of adults hospitalized for COVID-19 had an immunocompromising condition, demonstrating that this high-risk population continues to be overrepresented in hospital admissions nearly 2 years after the initial Omicron surge.^3,4^ This upward trend suggests that immunocompromised individuals now account for an even greater share of COVID-19 hospitalizations over time. These patients continue to suffer disproportionately worse outcomes. A 2025 analysis reported that immunocompromised individuals were 1.56 times more likely to require intensive care unit (ICU) admission and 1.64 times more likely to die in-hospital compared with immune-competent patients during the Omicron era.^5^ These findings clearly illustrate that immunosuppression remains a key driver of COVID-19 severity. Despite the overall decline in COVID-19 burden in the general population, morbidity and mortality remain substantial among immunocompromised individuals, including those with cancer, solid organ transplants, and hematologic malignancies. These patients often exhibit attenuated vaccine responses and prolonged viral shedding, highlighting their sustained vulnerability and the urgent need for timely antiviral interventions.^6-9^

Despite their elevated risk profile, immunocompromised individuals have been underrepresented in randomized controlled trials (RCTs) assessing COVID-19 treatments. As a result, there is limited high-certainty evidence to guide therapeutic decision-making in this group. Hospital protocols and national guidelines, often designed around data from immunocompetent populations, may offer incomplete or inconsistent recommendations for managing hospitalized patients with impaired immunity.^10,11^ In the absence of clear, up-to-date direction, treatment decisions for this group often default to clinical practice judgment. Today, clinical pharmacy teams, particularly those embedded within hospitals, play a central role in interpreting evolving evidence, implementing treatment protocols, and overseeing the appropriate utilization of antimicrobial and antiviral agents.

Among approved inpatient antivirals, remdesivir maintains the strongest evidence base when administered early in hospitalization. Real-world studies have recently shown that early remdesivir use may reduce mortality among immunocompromised patients,^12^ but these findings have not been widely incorporated into pharmacy practice or local guidelines. In a context where protocols have stagnated, clinical pharmacists need updated, patient-level data to justify and support appropriate and timely antiviral use.

This study builds on previous real-world analyses^13^ by extending the study period through December 2024, leveraging nearly 3 years of Omicron-era data, including both early and later Omicron periods, to evaluate the effectiveness of early remdesivir initiation in hospitalized immunocompromised patients. By capturing outcomes nearly a year beyond the end of the US public health emergency, this study provides updated evidence for a patient population that continues to face elevated risk.

## Methods

### Study design and data source

This retrospective cohort study assessed the effectiveness of early inpatient remdesivir use among immunocompromised adults hospitalized for COVID-19. Analyses were conducted using data from the Premier Healthcare Database (PHD; www.premierinc.com), a large, deidentified US hospital database that includes approximately 1 in 4 inpatient discharges nationwide. The database contains anonymized patient-level records, including demographics, diagnoses, procedures, medications, and hospital characteristics, enabling granular assessments of treatment patterns and outcomes.

The study period encompassed admissions from December 1, 2021, to December 31, 2024, covering the Omicron-predominant period, including its subvariant waves. This study replicated the study design and analytic approach used in a prior study evaluating a shorter timeframe from the same database.^13^

### Study population

The study population included adult patients (aged ≥18 years) hospitalized with a primary discharge diagnosis of COVID-19, defined by International Classification of Diseases, 10th Revision, Clinical Modification (ICD-10-CM) code U07.1, with a coding indication that the condition was present on admission. The analysis focused on patients with underlying immunocompromising conditions, identified through ICD-10-CM diagnosis codes for solid tumors, hematologic malignancies, solid organ or hematopoietic stem cell transplants, moderate to severe primary immunodeficiencies, HIV, immunosuppressive drug use, bone marrow failure syndromes, and other relevant disorders, consistent with the design of the previous study.^13^ Patients were excluded if they were pregnant, had incomplete hospitalization records, were transferred to or from other acute care facilities, transferred from hospice facilities, received extracorporeal membrane oxygenation on admission (during the first 2 days of hospitalization), died or were discharged within the first 2 days of hospitalization, or were initiated on remdesivir therapy after day 2 of hospitalization. To ensure accurate classification of oxygen status, only patients hospitalized at facilities affirmatively determined to charge separately for supplemental oxygen (ie, separate from daily room charges) were included. This allowed the absence of supplemental oxygen charges (no supplemental oxygen charges [NSOc]) to serve as a valid surrogate indicator for the absence of supplemental oxygen use.

### Treatment exposure, outcomes, and covariates

Patients were assigned to the treatment group based on whether they received at least 1 dose of remdesivir within the first 2 days following hospital admission. Patients who did not receive remdesivir during their hospitalization served as the comparator group. Those initiated on treatment after hospital day 2 despite coding of COVID-19 as being present on admission were excluded to reduce bias related to delayed or rescue therapy. To treat both groups equally and ensure no immortal time bias arises, patients that were in the hospital for less than 2 days were excluded, including both early discharges and early deaths.

The study endpoint was all-cause inpatient mortality, assessed at 14 and 28 days from a common start of follow-up at hospital day 3. Deaths were identified through discharge status codes, including discharges to hospice care. Patients discharged alive before the outcome assessment window were censored at 14 and 28 days, respectively.

Baseline covariates included demographic variables (age, gender, and race/ethnicity), comorbidities (eg, cardiovascular disease, diabetes mellitus, chronic kidney disease), specific immunocompromising conditions, hospital characteristics (eg, size, teaching status), payor type, and respiratory support intensity during the first 2 hospital days (supplementary eTable 1). Patients were stratified by oxygen use into 2 cohorts: those with NSOc and those who received any supplemental oxygen, including low-flow, high-flow, noninvasive ventilation, or mechanical ventilation. Supplemental oxygenation status was determined as the highest level of support recorded in the billing records within the first 2 days of the hospitalization, ie, the same time period during which treatment with remdesivir was identified. Further, in addition to supplemental oxygen requirements at baseline, acute illness severity upon admission was assessed based on the hospital ward in which the patient was admitted in the first 2 days of hospitalization (general ward vs ICU/step-down unit). Additional variables included use of concomitant COVID-19 therapies such as corticosteroids, interleukin-6 (IL-6) receptor inhibitors, and Janus kinase (JAK) inhibitors during the first 2 days of hospitalization.

### Statistical analysis

To mitigate confounding due to nonrandom treatment allocation, 1:1 propensity score (PS) matching without replacement was applied to match remdesivir recipients to nonrecipients. The PS was estimated using a logistic regression model, incorporating demographic and clinical characteristics, comorbidities, hospital characteristics, and calendar month of admission. PS matching was performed within predefined age groups and admission month strata and, when possible, within the same facility. A caliper of 0.2 standard deviation of the logit of the PS was applied. A detailed description of the methodology has been published previously.^13^ Postmatching balance was evaluated using standardized mean differences (SMDs), with a threshold of an absolute SMD of <0.15 considered acceptable.

Crude mortality rates were calculated, and Cox proportional hazards models were used to estimate adjusted hazard ratios (aHRs) and 95% confidence intervals (CIs) for the 14- and 28-day mortality outcomes. Models included robust sandwich estimators to account for clustering at the hospital level and were further adjusted for time-varying treatment with other COVID-19 medications (baricitinib, tocilizumab, and oral antivirals).

We conducted predefined subgroup analyses for patients with cancer (including hematologic malignancies), patients with hematologic malignancies alone, and those who had undergone solid organ or stem cell transplantation. These subgroups were not mutually exclusive. These subgroups were assessed using stabilized inverse probability of treatment weighting (IPTW) given the smaller sample size in each subgroup. Specific subgroup analyses for the hematologic malignancies of lymphoma and multiple myeloma and for solid organ or stem cell transplantation were exploratory given the small sample sizes in these categories.

Sensitivity analyses were performed to test robustness, including use of alternative definitions of early remdesivir initiation (initiation of remdesivir in the first 2 days vs no remdesivir in the first 2 days, allowing for subsequent crossover, analogous to an intention-to-treat analysis for a randomized controlled trial) and the alternative statistical approach of stabilized IPTW.

This study was reviewed by Advarra Institutional Review Board (protocol #Pro00091215) and was determined to be exempt from IRB oversight in accordance with the Department of Health and Human Services regulations found at 45 CFR 46.104(d)(4).

## Results

### Study population and cohort selection

**Figure 1.**
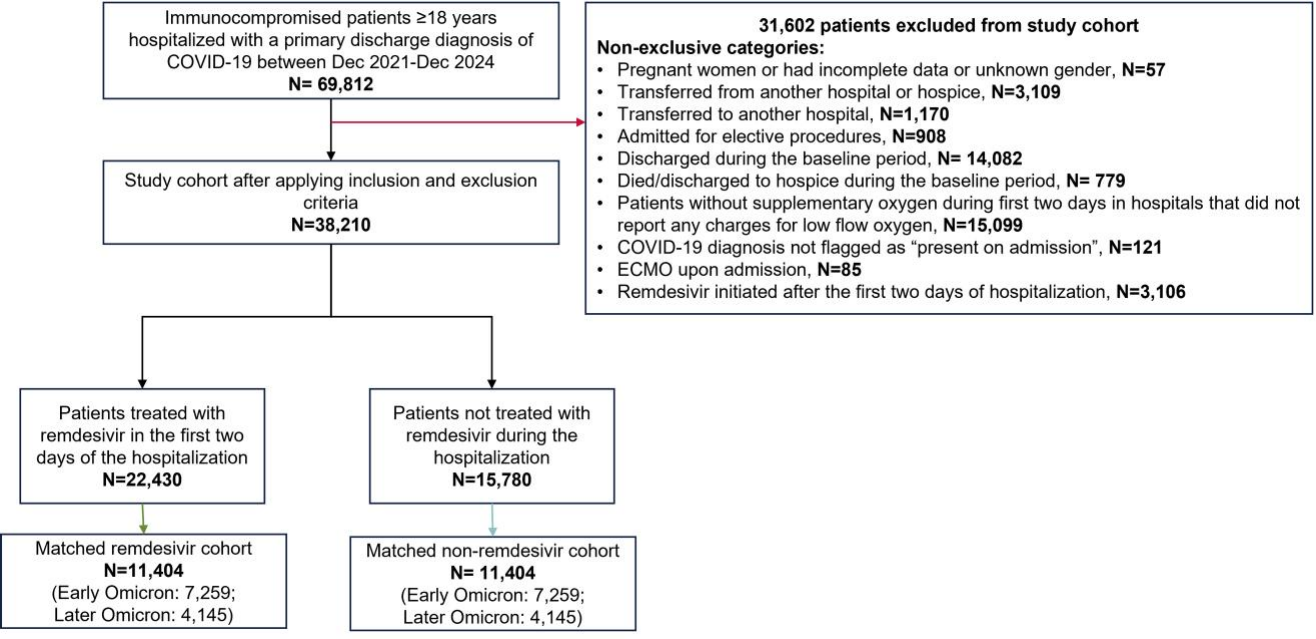
Study flow diagram. COVID-19 indicates coronavirus disease 2019; ECMO, extracorporeal membrane oxygenation

A total of 69,812 immunocompromised adult patients hospitalized with a primary discharge diagnosis of COVID-19 from December 2021 to December 2024 were initially identified. After applying exclusion criteria (Figure 1), the final study cohort included 38,210 patients. Specifically, 15,099 patients were excluded due to restricting analyses only to hospitals that reported any billing for supplementary oxygen. In addition, 779 patients were excluded due to early deaths (within the first 2 days of hospitalization), and thus mortality rates due to the most severe cases are unlikely to be underestimated in this study.

Among the final study cohort of 38,210 patients, 22,430 patients received remdesivir within the first 2 days of hospitalization, while 15,780 patients did not receive remdesivir during their hospital stay. PS matching without replacement yielded 2 balanced cohorts of 11,404 patients each (remdesivir-treated and untreated), which comprised the analysis population for this study (Figure 1).

### Baseline characteristics before and after matching

Baseline characteristics of the remdesivir-treated and untreated groups before and after PS matching are presented in the supplementary eTable 2. After matching, all covariates were well balanced, with absolute SMDs less than 0.15, including age distribution, gender, race, ethnicity, payor type, admission month, hospital characteristics, comorbidities, immunocompromising conditions, baseline supplemental oxygen requirements, and other treatments at baseline.

### Clinical outcomes

**Figure 2.**
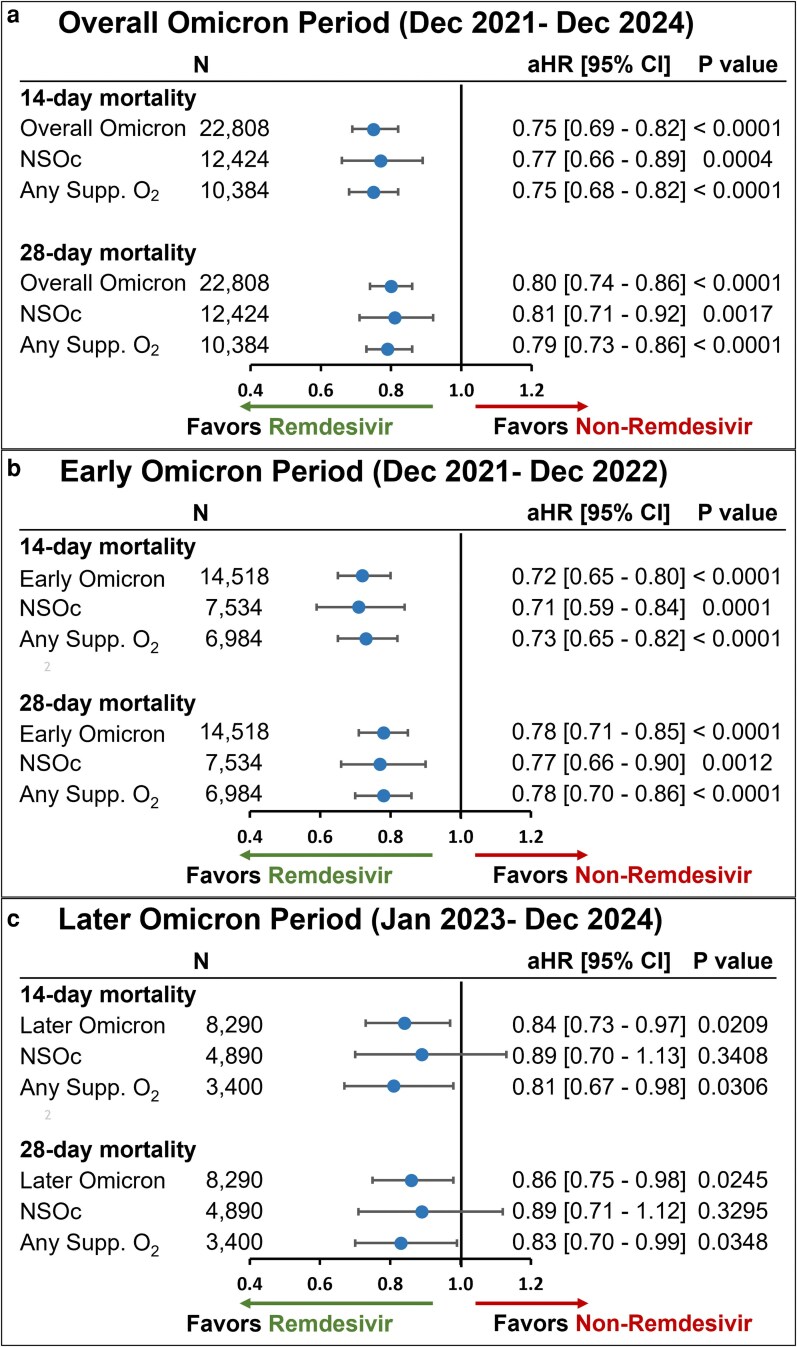
14- and 28-day mortality in immunocompromised patients hospitalized for coronavirus disease 2019 (COVID-19) present on admission and treated with remdesivir within the first 2 days of hospitalization versus those not treated with remdesivir during hospitalization, by baseline supplemental oxygen requirements, as determined in a propensity score matching analysis. A Cox proportional hazards model was used to derive estimates adjusted for admission month and time-varying treatment with other COVID-19 medications (baricitinib, tocilizumab, and oral antivirals) for (a) the overall Omicron period, (b) the early Omicron period, and (c) the later Omicron period. aHR indicates adjusted hazard ratio; CI, confidence interval; NSOc, no supplemental oxygen charges; Supp. O_2_, supplemental oxygen.

The unadjusted all-cause inpatient mortality rate in the crude population prior to PS matching was consistently lower in the remdesivir group versus the nonremdesivir group in the overall Omicron group as well as the NSOc and any supplemental oxygen subgroups (supplementary eTable 3). Post PS matching, this mortality benefit in the remdesivir group remained consistent. Among 22,808 PS-matched immunocompromised patients, the unadjusted all-cause inpatient mortality rate at 14 days was 9.0% in the remdesivir group versus 11.7% in the nonremdesivir group, while at 28 days, the mortality rate was 12.3% versus 15.0%, respectively (supplementary eTable 4). Early remdesivir initiation (within the first 2 days of hospitalization for patients admitted for COVID-19 present on admission) was associated with significantly lower all-cause mortality compared with no remdesivir use. At 14 days, mortality was reduced by 25% (HR, 0.75; 95% CI, 0.69-0.82; *P* < 0.0001), and at 28 days by 20% (HR, 0.80; 95% CI, 0.74-0.86; *P* < 0.0001). These associations remained consistent during both early and later Omicron periods (Figure 2).

Benefits were also observed across clinical subgroups. Among 12,424 matched patients with NSOc in the first 2 days, the unadjusted all-cause inpatient mortality rate at 14 days was 5.8% in the remdesivir group versus 7.4% in the non-remdesivir group, while at 28 days, the mortality rate was 7.7% versus 9.2%, respectively (supplementary eTable 4). Early remdesivir use was associated with reduced mortality (14-day HR, 0.77; 95% CI, 0.66-0.89; *P* = 0.0004; 28-day HR, 0.81; 95% CI, 0.71-0.92; *P* = 0.0017). Effect sizes for the NSOc subgroup were stronger in the early Omicron period but not statistically significant in the later period (Figure 2).

In the subgroup receiving any supplemental oxygen during the first 2 days (n = 10,384), the unadjusted all-cause inpatient mortality rate at 14 days was 12.9% in the remdesivir group versus 16.8% in the non-remdesivir group, while at 28 days, the mortality rate was 17.8% versus 21.9%, respectively (supplementary eTable 4). Early remdesivir was similarly associated with reduced 14-day (HR, 0.75; 95% CI, 0.68-0.82; *P* < 0.0001) and 28-day (HR, 0.79; 95% CI, 0.73-0.86; *P* < 0.0001) mortality, with consistent effects across variant periods (Figure 2).

### Sensitivity analyses

Consistent results were observed in 2 sensitivity analyses: one using stabilized IPTW and another comparing remdesivir initiation within the first 2 days of hospitalization to no remdesivir initiation within the first 2 days (including patients who crossed over to be initiated on remdesivir after the first 2 days of hospitalization) (supplementary eTable 5, eTable 6 and eTable 7).

### Subgroup analyses focused on specific immunocompromised conditions

**Figure 3.**
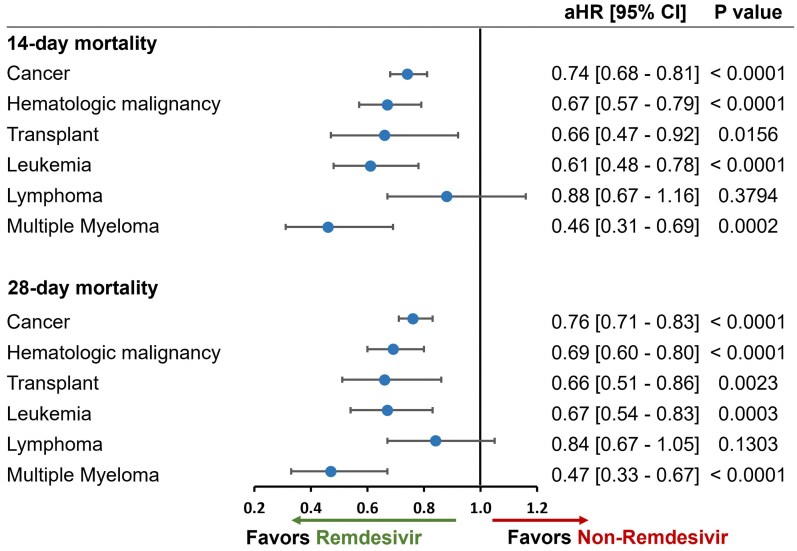
14- and 28-day mortality in immunocompromised patients hospitalized for coronavirus disease 2019 (COVID-19) present on admission and treated with remdesivir within the first 2 days of hospitalization versus those not treated with remdesivir during hospitalization, by subgroups of immunocompromising conditions, as determined by stabilized inverse probability of treatment weighting. A Cox proportional hazards model was used to derive estimates adjusted for admission month and time-varying treatment with other COVID-19 medications (baricitinib, tocilizumab, and oral antivirals). aHR indicates adjusted hazard ratio; CI, confidence interval.

In predefined immunocompromised subgroups evaluated using stabilized IPTW, early remdesivir use was associated with lower 14-day mortality in patients with cancer (HR, 0.74; 95% CI, 0.680-0.81), hematologic malignancies (HR, 0.67; 95% CI, 0.57-0.79), leukemia (HR, 0.61; 95% CI, 0.48-0.78), transplant (HR, 0.66; 95% CI, 0.47-0.92), and multiple myeloma (HR, 0.46; 95% CI, 0.31-0.69). No significant effect was observed for patients with lymphoma (HR, 0.88; 95% CI, 0.67-1.16). Findings were consistent at 28 days, with significant reductions in all subgroups except the lymphoma subgroup (Figure 3). The sample size of the lymphoma subgroup (∼600 patients in each treatment group) is not sufficient to detect significant differences at the observed HR ranging from 0.84 to 0.88 with the underlying overall event rate of ∼11%-13% for 14-day mortality and ∼16%-19% for 28-day mortality.

## Discussion

COVID-19 continues to represent a serious and potentially fatal threat for high-risk immunocompromised patients, despite the broader public misperceptions that SARS-CoV-2 no longer causes fatalities.^14^ In this large, real-world cohort of immunocompromised adults hospitalized for COVID-19 during the Omicron-dominant period, initiation of remdesivir within the first 2 days of hospitalization was associated with a significant reduction in 14- and 28-day all-cause mortality when compared to no remdesivir treatment. These associations were consistent across most subgroups, including patients with cancer and hematologic malignancies, patients who were transplant recipients, and those requiring supplemental oxygen at baseline. The clinical association of early initiation of remdesivir persisted throughout both the early and later Omicron period subvariant waves, when BQ.1, XBB.1.5, and JN.1 were the predominant circulating variants in the US, reflecting the sustained potential effectiveness of remdesivir in this vulnerable population.

As observed in this study, early remdesivir use was associated with benefits across clinical subgroups, including patients admitted without documented supplemental oxygen charges during the first 2 days of hospitalization. This finding is in line with the evidence from studies conducted during earlier stages of the pandemic, which reported a preserved relative risk reduction in mortality among patients admitted with NSOc.^15^ However, given the lower absolute mortality risk in the later Omicron period, the analysis of this subgroup may have been underpowered for a definitive statistical assessment. Considering the full temporal progression during the now endemic era of SARS-CoV-2, and the totality of the evidence across clinical trials and real-world studies, we suggest consideration of the early use of remdesivir, even for patients with no supplemental oxygen requirement.

Clinically, our study highlights the potential role of early antiviral therapy in improving survival outcomes among immunocompromised patients, who often experience impaired viral clearance and prolonged viral shedding. The observed mortality reductions with early remdesivir initiation, even in patients not requiring supplemental oxygen at baseline, support updated treatment strategies advocating for early intervention before respiratory compromise ensues. This evidence sits within a continuum of data, including findings from an RCT^16^ and real-world evidence, supporting the use of remdesivir in patients with 1 or more risk factors, to prevent progression to hospitalization^17^; and for hospitalized patients (with or without risk factors), to mitigate and decrease the ensuing risk of morbidity and mortality. Importantly, remdesivir’s efficacy appeared to be maintained during the later Omicron period, consistent with in vitro data demonstrating its potent antiviral activity and clinical observations confirming sustained effectiveness in the real-world setting.^18–20^ The evolving nature of the COVID-19 pandemic underscores the critical importance of real-world data to complement RCTs. While RCTs provide controlled evidence of efficacy, real-world data captures the effectiveness of interventions across diverse patient populations, including high-risk and underrepresented groups like immunocompromised patients, under real clinical conditions. This study’s findings reinforce and extend RCT evidence by demonstrating remdesivir’s sustained survival associations throughout the Omicron subvariant waves, reflecting its practical impact in routine hospital care. Such robust real-world insights are essential for informing timely clinical decisions and updating treatment protocols amid an evolving viral landscape.^13^

From a health-systems and clinical pharmacy perspective, these findings carry operational applicability. With the end of the public health emergency, the responsibility for ensuring evidence-based treatment of hospitalized COVID-19 patients has shifted almost entirely to inpatient care providers, including clinical pharmacy teams. Institutional treatment protocols differ across hospitals, and ongoing updates provide an opportunity to align practice with the latest evidence and patient needs. Clinical pharmacy teams are uniquely positioned to support this process. Pharmacists embedded in hospitals are responsible not only for interpreting evidence but also for managing protocols or developing order sets, overseeing stewardship, and ensuring appropriate patients receive timely therapy. With nearly one-third of apparently treatment-eligible immunocompromised COVID-19 patients not receiving remdesivir in our sample, our data suggests that even now, hospitalized immunocompromised patients meeting guideline-based criteria for early remdesivir are still not receiving this effective treatment. Looking forward, clinical pharmacists and hospital-based care teams should take proactive steps to ensure that emerging real-world evidence translates into updated, practical treatment guidance. Ongoing surveillance of antiviral effectiveness is essential as SARS-CoV-2 continues to evolve. Above all, pharmacy-led interventions, such as protocol updates, provider education, and real-time stewardship, will be crucial to closing the gap between evolving evidence and bedside practice. These actions are necessary to ensure that vulnerable patients receive the full potential benefit of proven therapies like remdesivir, thereby potentially reducing the risk of morbidity and mortality.

Our study has several strengths. We leveraged a large, geographically diverse national database representing approximately 25% of US hospitalizations, allowing for broad generalizability. The use of rigorous PS matching and IPTW minimized confounding and enhanced the robustness of associative findings. Comprehensive subgroup analyses provided insights into specific immunocompromised populations at particularly high risk. Additionally, by excluding patients who were initiated on remdesivir after the first 2 hospital days, we reduced bias from late (salvage) treatment initiation related to clinical deterioration or other confounders.

Nonetheless, limitations inherent to observational studies remain. Residual confounding cannot be fully excluded despite adjustment for numerous clinical and hospital-level covariates. Our reliance on billing and administrative data may introduce misclassification bias, particularly for oxygen use and comorbidities. Further, the reliance on billing data to identify oxygen use may reduce generalizability in other healthcare systems. The absence of specific laboratory data, including viral load or immune status markers, limits nuanced understanding of disease severity and response to treatment. Further, given that the database used is an inpatient chargemaster (billing) database, the patient record begins on the first day of the hospitalization. As such, only the secondary discharge diagnoses that were reported during the COVID-19 hospitalization (with COVID-19 being the primary discharge diagnosis) were utilized to identify the immunocompromising conditions of interest. We were unable to specify whether these were active conditions or a history of these conditions accurately; however, these conditions were critical enough to be reported during the COVID-19 hospitalization. Lastly, the database did not capture outpatient antiviral or monoclonal antibody use or vaccination status, which could potentially influence outcomes. Finally, the results presented in this paper focus on a distinct group of immunocompromised patients. For benefits of remdesivir in a broader population of hospitalized patients, see the article by Kalil et al (published as part of this supplement).

## Conclusions

Despite the waning public urgency around COVID-19, our extended real-world evidence suggests that COVID-19 continues to be associated with a mortality risk to immunocompromised hospitalized patients. Early remdesivir initiation was associated with significant survival benefits across multiple high-risk subgroups well beyond the end of the US public health emergency. These findings highlight the potential importance of early antiviral treatment in this vulnerable population, though further prospective studies, including RCTs, are needed to confirm the causal impact and optimize treatment strategies.

As institutional protocols evolve, clinical pharmacy teams and hospital care providers play a central, pivotal role in identifying at-risk patients, updating pharmacy order sets, and ensuring timely, evidence-informed antiviral clinical utilization. Ongoing engagement from inpatient care providers will be essential to ensure that institutional protocols evolve in alignment with the evidence and that immunocompromised patients hospitalized for SARS-CoV-2 infection consistently receive therapies that are associated with improved outcomes.

## Supplementary Material

zxag036_Supplementary_Data

## Data Availability

The data supporting this study’s findings are available from Premier, Inc. (https://www.premierinc.com/). Restrictions apply to the availability of these data, which were used under license for this study.

## Additional information

The authors provided the following information regarding their contributions to this work: All authors contributed substantially to the manuscript and agreed to the final submitted version.
