# Supplementary material for: Real-world effectiveness of remdesivir in immunocompromised patients hospitalized due to SARS-CoV-2 Infection: Insights to inform pharmacy practice
Source: Am J Health Syst Pharm. 2026 Feb 9;83(Suppl 3):S2931–9. doi: 10.1093/ajhp/zxag036 (PMC13070692; doi:10.1093/ajhp/zxag036)
Supplement: zxag036_Supplementary_Data [file zxag036_supplementary_data.docx]

## eTable 1. Definitions of Key Study Variables

| Key Study Variables | Definitions |
| --- | --- |
| Remdesivir treatment | Billing charges for treatment: Remdesivir;  ICD-10 procedure codes: XW033E5, XW043E5 |

| Immunocompromising conditions | Cancer | ICD-10-CM diagnosis codes: C00.x-C96.x |
| --- | --- | --- |
|  | Hematologic malignancy | ICD-10-CM diagnosis codes: C81.x, C82.x, C83.x, C84.x, C85.x, C88.x, C90.x, C91.x, C92.x, C93.x, C94.x, C95.x, C96.x |
|  | Leukemia | C91.x, C92.x, C93.x, C94.x, C95.x |
|  | Lymphoma | C81.x C82.x, C83.x, C84.x, C85.x |
|  | Multiple myeloma | C90.x |
|  | Solid organ/hematopoietic stem cell transplant recipients | ICD-10-CM diagnosis codes: Z94.x |
|  | Moderate or severe primary immunodeficiencies (example, severe combined immunodeficiencies, Ataxia-telangiectasia, Chediak-Higashi syndrome, Chronic granulomatous disease, Neutropenia) | ICD-10-CM diagnosis codes: D80.x, D81.x, D82.x, D83.x, D84.x, D86.x, D89.0, D89.1, D89.2, D89.3, D89.4x, D89.81, D89.82, D89.89, D89.9, G11.3, E70.330, D71.x, D70.x |
|  | Immunosuppressive medications (example, current use of systemic steroids, immunomodulator, immunosuppressant, chemotherapeutic agent, myelosuppressive agent) | ICD-10-CM diagnosis codes: Z79.52, Z79.60, Z79.61, Z79.62x, Z79.63x, Z79.64, Z79.69, Z79.81x |
|  | Asplenia | ICD-10-CM diagnosis codes: Q89.01, Z90.81 |
|  | Bone Marrow Failure/Aplastic anemia | ICD-10-CM diagnosis codes: D61.x |
|  | HIV | ICD-10-CM diagnosis codes: B20 |
|  | Toxic effects of antineoplastics | ICD-10-CM diagnosis codes: T45.1x |

| Key Comorbidities | Obesity | ICD-10-CM diagnosis codes: E66, Z68.25-Z68.45 |
| --- | --- | --- |
|  | Chronic Pulmonary Disease | ICD-10-CM diagnosis codes: I27.8, I27.9, J40, J41.x, J42, J43.x, J44.x, J45.x, J46, J47.x, J60, J61, J62.x, J63.x, J64, J65, J66.x, J67.x, J68.4, J70.1, J70.3 |
|  | Cardiovascular disease (including hypertension) | ICD-10-CM diagnosis codes: I00.x-I99.x |
|  | Diabetes Mellitus | ICD-10-CM diagnosis codes: E10.x-E14.x |
|  | Renal disease | ICD-10-CM diagnosis codes: I12.0, I13.1, N03.2, N03.3, N03.4, N03.5, N03.6, N03.7, N05.2, N05.3, N05.4, N05.5, N05.6, N05.7, N18.x, N19.x, N25.0, Z49.0, Z49.1, Z49.2, Z94.0, Z99.2 |
|  | Cancer | ICD-10-CM diagnosis codes: C00.x -C96.x |
| Supplemental oxygen requirements | IMV | Billing charges for devices: invasive mechanical ventilation, tracheostomy, endotracheal tube intubation, |
|  | ECMO | Billing charges for devices: extracorporeal membrane oxygenation |
|  | HFO/NIV | Billing charges for devices: negative-pressure ventilation, positive-pressure ventilation, CPAP, BiPAP, high flow system via nasal cannula, venturi face mask, rebreather, non-rebreather mask, positive expiratory pressure |
|  | LFO | Billing charges for devices/oxygen supply: simple face mask, oxygen pendant, low-flow system via nasal cannula, oxygen supply |
|  | NSOc | No billing charges for IMV, ECMO, HFO/NIV, or LFO at baseline |
| Admitting Diagnosis | Sepsis | ICD-10-CM diagnosis codes: A02.1, A32.7, A40.x, A41.x, A42.7, A54.86, B37.7, R65.20, R65.21, T81.44X, |
|  | Pneumonia | ICD-10-CM diagnosis codes: J12.x, J13, J14, J15.x, J16.x, J17, J18.x, A48.1, B25.0, A37.01, A37.11, A37.81, A37.91, A22.1, B44.0, B77.81, J10.00, J10.01, J10.08, J11.00, J11.08 |
| Baseline Medications | Anticoagulants | Billing charges for treatment at baseline: apixaban, argatroban, desirudin, lepirudin, dabigatran, danaparoid, edoxaban, tinzaparin, heparin (excluding use of heparin flush), ardeparin, bivalirudin |
|  | Corticosteroids | Billing charges for treatment at baseline: prednisone, prednisolone, methylprednisolone, hydrocortisone, dexamethasone |
|  | Convalescent plasma | Billing charges for treatment at baseline: convalescent plasma; ICD-10 procedure codes: XW13325, XW14325 |
|  | Tocilizumab | Billing charges for treatment at baseline: tocilizumab; ICD-10 procedure codes: XW033H5, XW043H5 |
|  | Baricitinib | Billing charges for treatment at baseline: Baricitinib; ICD-10 procedure codes: XW0DXM6, XW0H7M6, XW0G7M6 |
|  | Oral antivirals | Billing charges for treatment at baseline: nirmatrelvir–ritonavir, molnupiravir |
| Abbreviations: BiPAP, bilevel positive airway pressure; CPAP, continuous positive airway pressure; COPD, chronic obstructive pulmonary disease; HFO/NIV, high flow oxygen/non-invasive ventilation; HIV, human immunodeficiency virus; ICD-10-CM, International Classification of Diseases, 10th Revision (Clinical Modification); LFO, low flow oxygen; IMV, invasive mechanical ventilation; ECMO, extracorporeal membrane oxygenation; NSOc, no supplemental oxygen charges | | |

## eTable 2. Baseline Characteristics of Immunocompromised Patients Hospitalized For COVID-19 (December 2021 - December 2024), Before and After PS Matching

|  | **Before PS matching** | | | **After PS matching** | | |
| --- | --- | --- | --- | --- | --- | --- |
|  | **Remdesivir** | **Non-Remdesivir** | **SMD** | **Remdesivir** | **Non-Remdesivir** | **SMD** |
| **Number of patients** | **n=15,780** | **n=22,430** |  | **n=11,404** | **n=11,404** | **-** |
| **Age group, years** |  |  |  |  |  |  |
| 18–49 | 1,500 (6.7) | 1,032 (6.5) | 0.00 | 538 (4.7) | 538 (4.7) | 0.00 |
| 50–64 | 4,595 (20.5) | 3,106 (19.7) |  | 2,043 (17.9) | 2,043 (17.9) |  |
| ≥65 | 16,335 (72.8) | 11,642 (73.8) |  | 8,823 (77.4) | 8,823 (77.4) |  |
| **Race** |  |  |  |  |  |  |
| White | 17,007 (75.8) | 11,975 (75.9) | 0.09 | 8,833 (77.5) | 8,731 (76.6) | 0.00 |
| Black | 3,144 (14.0) | 2,548 (16.1) |  | 1,656 (14.5) | 1,724 (15.1) |  |
| Asian | 576 (2.6) | 256 (1.6) |  | 190 (1.7) | 206 (1.8) |  |
| Other | 1,703 (7.6) | 1,001 (6.3) |  | 725 (6.4) | 743 (6.5) |  |
| **Gender** |  |  |  |  |  |  |
| Female | 11,489 (51.2) | 8,003 (50.7) | 0.01 | 5,766 (50.6) | 5,768 (50.6) | 0.00 |
| **Ethnicity** |  |  |  |  |  |  |
| Hispanic | 2,524 (11.3) | 1,207 (7.6) | 0.11 | 930 (8.2) | 914 (8.0) | 0.00 |
| Non-Hispanic | 18,534 (82.6) | 13,389 (84.8) |  | 9,674 (84.8) | 9,710 (85.1) |  |
| Unknown | 1,372 (6.1) | 1,184 (7.5) |  | 800 (7.0) | 780 (6.8) |  |
| **Primary payor** |  |  |  |  |  |  |
| Commercial | 3,074 (13.7) | 1,888 (12.0) | 0.09 | 1,283 (11.3) | 1,312 (11.5) | 0.06 |
| Medicare | 16,902 (75.4) | 12,156 (77.0) |  | 8,983 (78.8) | 9,000 (78.9) |  |
| Medicaid | 1,704 (7.6) | 1,106 (7.0) |  | 717 (6.3) | 699 (6.1) |  |
| Other | 750 (3.3) | 630 (4.0) |  | 421 (3.7) | 393 (3.4) |  |
| **Admission source** |  |  |  |  |  |  |
| Transfer from SNF or ICF | 776 (3.5) | 506 (3.2) | 0.01 | 381 (3.3) | 382 (3.3) | 0.00 |
| **Hospital bed size** |  |  |  |  |  |  |
| <100 | 1,414 (6.3%) | 1,021 (6.5) | 0.16 | 735 (6.4) | 720 (6.3) | 0.03 |
| 100–199 | 3,419 (15.2%) | 2,652 (16.8) |  | 1,687 (14.8) | 1,702 (14.9) |  |
| 200–299 | 4,141 (18.5) | 3,110 (19.7) |  | 2,290 (20.1) | 2,333 (20.5) |  |
| 300–399 | 3,676 (16.4) | 3,010 (19.1) |  | 2,165 (19.0) | 2,162 (19.0) |  |
| 400–499 | 2,412 (10.8) | 1,813 (11.5) |  | 1,327 (11.6) | 1,287 (11.3) |  |
| ≥500 | 7,368 (32.8) | 4,174 (26.5) |  | 3,200 (28.1) | 3,200 (28.1) |  |
| **Hospital location** |  |  |  |  |  |  |
| Urban | 20,251 (90.3) | 13,873 (87.9) | 0.08 | 10,183 (89.3) | 10,158 (89.1) | 0.01 |
| Rural | 2,179 (9.7) | 1,907 (12.1) |  | 1,221 (10.7) | 1,246 (10.9) |  |
| **Teaching hospital** | 11,302 (50.4) | 7,193 (45.6) | 0.10 | 5,302 (46.5) | 5,323 (46.7) | 0.00 |
| **Region** |  |  |  |  |  |  |
| Midwest | 4,960 (22.1) | 3,830 (24.3) | 0.19 | 2,782 (24.4) | 2,791 (24.5) | 0.00 |
| Northeast | 4,105 (18.3) | 1,844 (11.7) |  | 1,538 (13.5) | 1,507 (13.2) |  |
| South | 10,810 (48.2) | 8,520 (54.0) |  | 5,927 (52.0) | 5,917 (51.9) |  |
| West | 2,555 (11.4) | 1,586 (10.1) |  | 1,157 (10.1) | 1,189 (10.4) |  |
| **Comorbid conditions** |  |  |  |  |  |  |
| Obesity | 5,642 (25.2) | 3,919 (24.8) | 0.01  0.09 | 2,780 (24.4) | 2,819 (24.7) | 0.01  0.00 |
| Chronic Pulmonary Disease | 9,533 (42.5) | 6,020 (38.1) |  | 4,599 (40.3) | 4,576 (40.1) |  |
| Cardiovascular disease | 19,986 (89.1) | 14,241 (90.2) | 0.04 | 10,323 (90.5) | 10,286 (90.2) | 0.01 |
| Diabetes Mellitus | 8,469 (37.8) | 6,119 (38.8) | 0.02 | 4,369 (38.3) | 4,353 (38.2) | 0.00 |
| Renal disease | 7,281 (32.5) | 6,270 (39.7) | 0.15 | 4,227 (37.1) | 4,246 (37.2) | 0.00 |
| Cancer | 9,625 (42.9) | 6,603 (41.8) | 0.02 | 4,841 (42.5) | 4,873 (42.7) | 0.01 |
| **Immunocompromising condition** | 22,430 (100.0) | 15,780 (100.0) | 0.00 | 11,404 (100.0) | 11,404 (100.0) | 0.00 |
| **Hospital ward upon admission** |  |  |  |  |  |  |
| General ward | 18,230 (81.3) | 13,363 (84.7) | 0.09 | 9,687 (84.9) | 9,670 (84.8) | 0.00 |
| ICU/Step down unit | 4,200 (18.7) | 2,417 (15.3) |  | 1,717 (15.1) | 1,734 (15.2) |  |
| **Admission diagnosis** |  |  |  |  |  |  |
| Sepsis | 121 (0.5) | 99 (0.6) | 0.01  0.00 | 59 (0.5) | 69 (0.6) | 0.01  0.00 |
| Pneumonia | 1,418 (6.3) | 1,014 (6.4) |  | 701 (6.1) | 693 (6.1) |  |
| **Other treatments at baseline** |  |  |  |  |  |  |
| Anticoagulants | 16,805 (74.9) | 10,759 (68.2) | 0.15 | 8,195 (71.9) | 8,207 (72.0) | 0.00 |
| Convalescent plasma | 47 (0.2) | 9 (0.1) | 0.38 | 6 (0.1) | 5 (0.0) | 0.01 |
| Corticosteroids | 18,948 (84.5) | 10,809 (68.5) | 0.04 | 8,995 (78.9) | 8,967 (78.6) | 0.00 |
| Baricitinib | 750 (3.3) | 594 (3.8) | 0.06 | 425 (3.7) | 416 (3.6) | 0.01 |
| Tocilizumab | 640 (2.9) | 315 (2.0) | 0.02 | 267 (2.3) | 250 (2.2) | 0.00 |
| Oral antivirals^1^ | 73 (0.3) | 403 (2.6) | 0.19 | 38 (0.3) | 37 (0.3) | 0.00 |
| **Baseline supplemental oxygen requirements** | | |  |  |  |  |
| NSOc | 10,669 (47.6) | 8,763 (55.5) | 0.19 | 6,212 (54.5) | 6,212 (54.5) | 0.00 |
| LFO | 7,039 (31.4) | 4,339 (27.5) |  | 3,357 (29.4) | 3,357 (29.4) |  |
| HFO/NIV | 4,229 (18.9) | 2,208 (14.0) |  | 1,656 (14.5) | 1,656 (14.5) |  |
| IMV | 493 (2.2) | 470 (3.0) |  | 179 (1.6) | 179 (1.6) |  |
| **Omicron period** |  |  |  |  |  |  |
| Early (Dec 2021-Dec 2022) | 12,845 (57.3) | 9,800 (62.1) | 0.10 | 7,259 (63.7) | 7,259 (63.7) | 0.00 |
| Later (Jan 2023-Dec 2024) | 9,585 (42.7) | 5,980 (37.9) |  | 4,145 (36.3) | 4,145 (36.3) |  |
| **Type of immunocompromising condition** | |  |  |  |  |  |
| Cancer | 9,625 (42.9) | 6,603 (41.8) | 0.02 | 4,841 (42.5) | 4,873 (42.7) | 0.01 |
| Hematologic malignancies | 3,540 (15.8) | 2,407 (15.3) | 0.01 | 1,832 (16.1) | 1,776 (15.6) | 0.01 |
| Leukemia | 1,357 (6.0) | 926 (5.9) | 0.01 | 724 (6.3) | 667 (5.8) | 0.02 |
| Lymphoma | 1,209 (5.4) | 825 (5.2) | 0.01 | 619 (5.4) | 608 (5.3) | 0.00 |
| Multiple Myeloma | 746 (3.3) | 510 (3.2) | 0.01 | 386 (3.4) | 384 (3.4) | 0.00 |
| Solid organ and hematopoietic stem cell transplant | 1,758 (7.8) | 1141 (7.2) | 0.02 | 827 (7.3) | 833 (7.3) | 0.00 |
| Moderate or severe primary immunodeficiencies | 7,049 (31.4) | 3,979 (25.2) | 0.14 | 3,144 (27.6) | 3,113 (27.3) | 0.01 |
| Immunosuppressive medications | 8,144 (36.3) | 5,305 (33.6) | 0.06 | 3,962 (34.7) | 3,957 (34.7) | 0.00 |
| Asplenia | 421 (1.9) | 344 (2.2) | 0.02 | 223 (2.0) | 235 (2.1) | 0.01 |
| Bone Marrow Failure/Aplastic anemia | 2,904 (12.9) | 2,577 (16.3) | 0.10 | 1,675 (14.7) | 1,646 (14.4) | 0.01 |
| Human immunodeficiency virus | 392 (1.7) | 291 (1.8) | 0.01 | 156 (1.4) | 180 (1.6) | 0.02 |
| Toxic effects of antineoplastics | 1,214 (5.4) | 804 (5.1) | 0.01 | 574 (5.0) | 583 (5.1) | 0.00 |
| Data is presented as n (%), unless otherwise indicated.  ^1^See Supplemental Table S2  Abbreviations: COVID-19, coronavirus disease 2019; HFO/NIV, High-flow oxygen/non-invasive ventilation; NSOc, No supplementary oxygen charges; ICU, Intensive Care Unit; IMV, Invasive mechanical ventilation; INF, intermediate care facility; IPTW, inverse probability treatment weighting; LFO, Low-flow oxygen; PS, propensity score; SMD, standardized mean difference; SNF, skilled nursing facility. | | | | | | |

**eTable 3. Unadjusted All-cause Inpatient Mortality Rates in the Overall Omicron Period (Crude Population prior to PS Matching)**

|  | **Overall Omicron** | | **NSOc** | | **Any Supplemental Oxygen** | |
| --- | --- | --- | --- | --- | --- | --- |
|  | **Non-RDV** | **RDV** | **Non-RDV** | **RDV** | **Non-RDV** | **RDV** |
| **Overall population** | **n=15780** | **n=22430** | **n=8763** | **n=10669** | **n=7017** | **n=11761** |
| 14-day crude mortality rate | 1772 (11.2) | 1973 (8.8) | 621 (7.1) | 577 (5.4) | 1151 (16.4) | 1396 (11.9) |
| 28-day crude mortality rate | 2275 (14.4) | 2673 (11.9) | 790 (9.0) | 770 (7.2) | 1485 (21.2) | 1903 (16.2) |

Data is presented as n (%) unless otherwise indicated.

Abbreviations: PS, propensity score; NSOc, no supplemental oxygen charges.

## eTable 4. Unadjusted All-cause Inpatient Mortality Rates (PS matching)

|  | **Early Omicron (Dec 2021 - Dec 2022)** | | **Later Omicron (Jan 2023- Dec 2024)** | | **Overall (Dec 2021- Dec 2024)** | |
| --- | --- | --- | --- | --- | --- | --- |
|  | **No Remdesivir during the hospitalization** | **Remdesivir in the first 2 days** | **No Remdesivir during the hospitalization** | **Remdesivir in the first 2 days** | **No Remdesivir during the hospitalization** | **Remdesivir in the first 2 days** |
| **Overall Omicron** | **n=7259** | **n=7259** | **n=4145** | **n=4145** | **n=11404** | **n=11404** |
| 14-day mortality | 965 (13.3) | 722 (9.9) | 365 (8.8) | 308 (7.4) | 1,330 (11.7) | 1,030 (9.0) |
| 28-day mortality | 1,270 (17.5) | 1,031 (14.2) | 436 (10.5) | 375 (9.0) | 1,706 (15.0) | 1,406 (12.3) |
| **NSOc** | **n=3767** | **n=3767** | **n=2445** | **n=2445** | **n=6212** | **n=6212** |
| 14-day mortality | 308 (8.2) | 229 (6.1) | 149 (6.1) | 132 (5.4) | 457 (7.4) | 361 (5.8) |
| 28-day mortality | 395 (10.5) | 325 (8.6) | 175 (7.2) | 155 (6.3) | 570 (9.2) | 480 (7.7) |
| **Any supplemental oxygen** | **n=3492** | **n=3492** | **n=1700** | **n=1700** | **n=5192** | **n=5192** |
| 14-day mortality | 657 (18.8) | 493 (14.1) | 216 (12.7) | 176 (10.4) | 873 (16.8) | 669 (12.9) |
| 28-day mortality | 875 (25.1) | 706 (20.2) | 261 (15.4) | 220 (12.9) | 1,136 (21.9) | 926 (17.8) |

Data is presented as n (%) unless otherwise indicated.

Abbreviations: PS, propensity score; NSOc, no supplemental oxygen charges.

## eTable 5. Baseline Characteristics of Immunocompromised Patients Hospitalized for COVID-19 During December 2021-December 2024, Before and After Stabilized IPTW

|  | **Before stabilized IPTW** | | | **After stabilized IPTW** | | |
| --- | --- | --- | --- | --- | --- | --- |
|  | **Remdesivir** | **Non-Remdesivir** | **SMD** | **Remdesivir** | **Non-Remdesivir** | **SMD** |
| **Number of patients** | **n=15780** | **n=22430** |  | **-** | **-** | **-** |
| **Age group, years** |  |  |  |  |  |  |
| 18–49 | 1,500 (6.7) | 1,032 (6.5) | 0.00 | 6.6 | 6.6 | 0.00 |
| 50–64 | 4,595 (20.5) | 3,106 (19.7) |  | 20.1 | 20.2 |  |
| ≥65 | 16,335 (72.8) | 11,642 (73.8) |  | 73.2 | 73.2 |  |
| **Race** |  |  |  |  |  |  |
| White | 17,007 (75.8) | 11,975 (75.9) | 0.09 | 75.8 | 75.9 | 0.00 |
| Black | 3,144 (14.0) | 2,548 (16.1) |  | 14.9 | 14.8 |  |
| Asian | 576 (2.6) | 256 (1.6) |  | 2.2 | 2.2 |  |
| Other | 1,703 (7.6) | 1,001 (6.3) |  | 7.1 | 7.2 |  |
| **Gender** |  |  |  |  |  |  |
| Female | 11,489 (51.2) | 8,003 (50.7) | 0.01 | 50.8 | 51.0 | 0.00 |
| **Ethnicity** |  |  |  |  |  |  |
| Hispanic | 2,524 (11.3) | 1,207 (7.6) | 0.11 | 9.7 | 9.8 | 0.00 |
| Non-Hispanic | 18,534 (82.6) | 13,389 (84.8) |  | 83.6 | 83.7 |  |
| Unknown | 1,372 (6.1) | 1,184 (7.5) |  | 6.7 | 6.6 |  |
| **Primary payor** |  |  |  |  |  |  |
| Commercial | 3,074 (13.7) | 1,888 (12.0) | 0.09 | 12.8 | 12.9 | 0.00 |
| Medicare | 16,902 (75.4) | 12,156 (77.0) |  | 76.1 | 76.0 |  |
| Medicaid | 1,704 (7.6) | 1,106 (7.0) |  | 7.4 | 7.4 |  |
| Other | 750 (3.3) | 630 (4.0) |  | 3.7 | 3.7 |  |
| **Admission source** |  |  |  |  |  |  |
| Transfer from SNF or ICF | 776 (3.5) | 506 (3.2) | 0.01 | 3.4 | 3.4 | 0.00 |
| **Hospital bed size** |  |  |  |  |  |  |
| <100 | 1,414 (6.3) | 1,021 (6.5) | 0.16 | 6.4 | 6.6 |  |
| 100–199 | 3,419 (15.2) | 2,652 (16.8) |  | 15.7 | 15.6 | 0.04 |
| 200–299 | 4,141 (18.5) | 3,110 (19.7) |  | 19.0 | 18.8 |  |
| 300–399 | 3,676 (16.4) | 3,010 (19.1) |  | 17.3 | 17.3 |  |
| 400–499 | 2,412 (10.8) | 1,813 (11.5) |  | 11.2 | 11.2 |  |
| ≥500 | 7,368 (32.8) | 4,174 (26.5) |  | 30.3 | 30.5 |  |
| **Hospital location** |  |  |  |  |  |  |
| Urban | 20,251 (90.3) | 13,873 (87.9) | 0.08 | 89.4 | 89.3 | 0.00 |
| Rural | 2,179 (9.7) | 1,907 (12.1) |  | 10.6 | 10.7 |  |
| **Teaching hospital** | 11,302 (50.4) | 7,193 (45.6) | 0.10 | 48.5 | 48.5 | 0.00 |
| **Region** |  |  |  |  |  |  |
| Midwest | 4,960 (22.1) | 3,830 (24.3) | 0.19 | 23.2 | 23.2 | 0.00 |
| Northeast | 4,105 (18.3) | 1,844 (11.7) |  | 15.5 | 15.7 |  |
| South | 10,810 (48.2) | 8,520 (54.0) |  | 50.3 | 50.2 |  |
| West | 2,555 (11.4) | 1,586 (10.1) |  | 10.9 | 10.9 |  |
| **Comorbid conditions** |  |  |  |  |  |  |
| Obesity | 5,642 (25.2) | 3,919 (24.8) | 0.01  0.09 | 25.0 | 25.2 | 0.00  0.00 |
| Chronic Pulmonary Disease | 9,533 (42.5) | 6,020 (38.1) |  | 40.9 | 41.1 |  |
| Cardiovascular disease | 19,986 (89.1) | 14,241 (90.2) | 0.04 | 89.5 | 89.6 | 0.00 |
| Diabetes Mellitus | 8,469 (37.8) | 6,119 (38.8) | 0.02 | 38.1 | 38.3 | 0.00 |
| Renal disease | 7,281 (32.5) | 6,270 (39.7) | 0.15 | 35.5 | 35.5 | 0.00 |
| Cancer | 9,625 (42.9) | 6,603 (41.8) | 0.02 | 42.2 | 42.4 | 0.00 |
| **Immunocompromising condition** | 22,430 (100.0) | 15,780 (100.0) | 0.00 | 100.0 | 100.0 | 0.00 |
| **Hospital ward upon admission** |  |  |  |  |  |  |
| General ward | 18,230 (81.3) | 13,363 (84.7) | 0.09 | 82.7 | 82.7 | 0.00 |
| ICU/Step down unit | 4,200 (18.7) | 2,417 (15.3) |  | 17.3 | 17.3 |  |
| **Admission diagnosis** |  |  |  |  |  |  |
| Sepsis | 121 (0.5) | 99 (0.6) | 0.01  0.00 | 0.6 | 0.6 | 0.00  0.00 |
| Pneumonia | 1,418 (6.3) | 1,014 (6.4) |  | 6.4 | 6.4 |  |
| **Other treatments at baseline** |  |  |  |  |  |  |
| Anticoagulants | 16,805 (74.9) | 10,759 (68.2) | 0.15 | 72.2 | 72.4 | 0.00 |
| Convalescent plasma | 47 (0.2) | 9 (0.1) | 0.38 | 0.1 | 0.1 | 0.00 |
| Corticosteroids | 18,948 (84.5) | 10,809 (68.5) | 0.04 | 78.3 | 78.3 | 0.00 |
| Baricitinib | 750 (3.3) | 594 (3.8) | 0.06 | 3.6 | 3.6 | 0.01 |
| Tocilizumab | 640 (2.9) | 315 (2.0) | 0.02 | 2.5 | 2.6 | 0.00 |
| Oral antivirals | 73 (0.3) | 403 (2.6) | 0.19 | 1.2 | 1.2 | 0.00 |
| **Baseline supplemental oxygen requirements** | | |  |  |  |  |
| NSOc | 10,669 (47.6) | 8,763 (55.5) | 0.19 | 50.5 | 50.4 | 0.00 |
| LFO | 7,039 (31.4) | 4,339 (27.5) |  | 29.9 | 29.7 |  |
| HFO/NIV | 4,229 (18.9) | 2,208 (14.0) |  | 16.9 | 17.3 |  |
| IMV | 493 (2.2) | 470 (3.0) |  | 2.6 | 2.6 |  |
| **Omicron period** |  |  |  |  |  |  |
| Early (Dec 2021-Dec 2022) | 12,845 (57.3) | 9,800 (62.1) | 0.10 | 59.2 | 59.2 | 0.00 |
| Late (Jan 2023-Dec 2024) | 9,585 (42.7) | 5,980 (37.9) |  | 40.8 | 40.8 |  |
| **Type of immunocompromising condition** | |  |  |  |  |  |
| Cancer | 9,625 (42.9) | 6,603 (41.8) | 0.02 | 57.8 | 57.6 | 0.00 |
| Hematologic malignancies | 3,540 (15.8) | 2,407 (15.3) | 0.01 | 84.4 | 84.3 | 0.00 |
| Leukemia | 1,357 (6.0) | 926 (5.9) | 0.01 | 94.1 | 94.0 | 0.00 |
| Lymphoma | 1,209 (5.4) | 825 (5.2) | 0.01 | 94.6 | 94.6 | 0.00 |
| Multiple Myeloma | 746 (3.3) | 510 (3.2) | 0.01 | 96.7 | 96.7 | 0.00 |
| Solid organ and hematopoietic stem cell transplant | 1,758 (7.8) | 1141 (7.2) | 0.02 | 92.4 | 92.4 | 0.00 |
| Moderate or severe primary immunodeficiencies | 7,049 (31.4) | 3,979 (25.2) | 0.14 | 70.9 | 70.8 | 0.00 |
| Immunosuppressive medications | 8,144 (36.3) | 5,305 (33.6) | 0.06 | 35.4 | 35.4 | 0.00 |
| Asplenia | 421 (1.9) | 344 (2.2) | 0.02 | 98.0 | 98.0 | 0.00 |
| Bone Marrow Failure/Aplastic anemia | 2,904 (12.9) | 2,577 (16.3) | 0.10 | 85.8 | 85.8 | 0.00 |
| Human immunodeficiency virus | 392 (1.7) | 291 (1.8) | 0.01 | 98.2 | 98.2 | 0.00 |
| Toxic effects of antineoplastics | 1,214 (5.4) | 804 (5.1) | 0.01 | 94.7 | 94.7 | 0.00 |
| Data is presented as n (%) before matching and as % after matching, unless otherwise indicated.  Abbreviations: COVID-19, coronavirus disease 2019; HFO/NIV, High-flow oxygen/non-invasive ventilation; NSOc, No supplementary oxygen charges; ICU, Intensive Care Unit; IMV, Invasive mechanical ventilation; INF, intermediate care facility; IPTW, inverse probability treatment weighting; LFO, Low-flow oxygen; SMD, standardized mean difference; SNF, skilled nursing facility. | | | | | | |

## eTable 6. 14- and 28-day Mortality in Immunocompromised Patients Hospitalized for COVID-19 Present-on-Admission Treated with Remdesivir Within the First 2 Days of Hospitalization vs Those Not Treated with Remdesivir during the Hospitalization (Stabilized IPTW)

Cox Proportional Hazards model used to derive estimates adjusted for admission month, and time-varying treatment with other COVID-19 medications (baricitinib, tocilizumab, oral antivirals). aHR indicates adjusted hazard ratio; CI, confidence interval; COVID-19, coronavirus disease 2019; IPTW, inverse probability of treatment weighting.

|  | **aHR [95% CI]** | ***P* value** |
| --- | --- | --- |
| **14-day mortality** |  |  |
| Overall Omicron (Dec 2021-Dec 2024) | 0.74 [0.69 - 0.80] | <0.0001 |
| Early Omicron (Dec 2021-Dec 2022) | 0.73 [0.67 - 0.79] | <0.0001 |
| Later Omicron (Jan 2023-Dec 2024) | 0.79 [0.70 - 0.89] | 0.0001 |
| **28-day mortality** |  |  |
| Overall Omicron (Dec 2021-Dec 2024) | 0.77 [0.72 - 0.83] | <0.0001 |
| Early Omicron (Dec 2021-Dec 2022) | 0.77 [0.71 - 0.83] | <0.0001 |
| Later Omicron (Jan 2023-Dec 2024) | 0.80 [0.72 - 0.90] | 0.0001 |

## eTable 7. 14- and 28-day Mortality in Immunocompromised Patients Hospitalized for COVID-19 Present-on-Admission Treated with Remdesivir Within the First 2 Days of Hospitalization vs Those Not Treated with Remdesivir Within the First 2 Days of Hospitalization (includes the late remdesivir start) (PS matching)

Cox Proportional Hazards model used to derive estimates adjusted for admission month, and time-varying treatment with other COVID-19 medications (baricitinib, tocilizumab, oral antivirals). aHR indicates adjusted hazard ratio; CI, confidence interval; COVID-19, coronavirus disease 2019; PS, propensity score.

|  | **N** | **aHR [95% CI]** | ***P* value** |
| --- | --- | --- | --- |
| **14-day mortality** |  |  |  |
| Overall Omicron (Dec 2021-Dec 2024) | 25,064 | 0.76 [0.70 - 0.82] | <0.0001 |
| Early Omicron (Dec 2021-Dec 2022) | 15,866 | 0.75 [0.68 - 0.82] | <0.0001 |
| Later Omicron (Jan 2023-Dec 2024) | 9,198 | 0.81 [0.71 - 0.93] | 0.0031 |
| **28-day mortality** |  |  |  |
| Overall Omicron (Dec 2021-Dec 2024) | 25,064 | 0.77 [0.72 - 0.83] | <0.0001 |
| Early Omicron (Dec 2021-Dec 2022) | 15,866 | 0.76 [0.70 - 0.83] | <0.0001 |
| Later Omicron (Jan 2023-Dec 2024) | 9,198 | 0.83 [0.73 - 0.94] | 0.0034 |
